# What is known about paediatric nurse burnout: a scoping review

**DOI:** 10.1186/s12960-020-0451-8

**Published:** 2020-02-11

**Authors:** Laura Buckley, Whitney Berta, Kristin Cleverley, Christina Medeiros, Kimberley Widger

**Affiliations:** 1 Lawrence S. Bloomberg Faculty of Nursing, University of Toronto, 155 College Street, Toronto, ON M5T 3M6 Canada; 2 Hospital for Sick Children, 555 University Avenue, Toronto, ON M5G 1X8 Canada; 3 Institute for Health Policy, Management and Evaluation, University of Toronto, 155 College Street, Toronto, ON M5T 3M6 Canada; 4 Margaret and Wallace McCain Centre for Child, Youth and Family Mental Health, Centre for Addiction and Mental Health, Toronto, ON M5T 1R8 Canada

**Keywords:** Burnout, Burn out, Work stress, Pediatric nurses, Pediatrics, Pediatrics, Nurses

## Abstract

Burnout in healthcare providers has impacts at the level of the individual provider, patient, and organization. While there is a substantial body of literature on burnout in healthcare providers, burnout in pediatric nurses has received less attention. This subpopulation may be unique from adult care nurses because of the specialized nature of providing care to children who are typically seen as a vulnerable population, the high potential for empathetic engagement, and the inherent complexities in the relationships with families. Thus, the aim of this scoping review was to investigate, among pediatric nurses, (i) the prevalence and/or degree of burnout, (ii) the factors related to burnout, (iii) the outcomes of burnout, and (iv) the interventions that have been applied to prevent and/or mitigate burnout. This scoping review was performed according to the PRISMA Guidelines Scoping Review Extension. CINAHL, EMBASE, MEDLINE, PsycINFO, ASSIA, and The Cochrane Library were searched on 3 November 2018 to identify relevant quantitative, qualitative, and mixed-method studies on pediatric nurse burnout. Our search identified 78 studies for inclusion in the analysis. Across the included studies, burnout was prevalent in pediatric nurses. A number of factors were identified as impacting burnout including nurse demographics, work environment, and work attitudes. Similarly, a number of outcomes of burnout were identified including nurse retention, nurse well-being, patient safety, and patient-family satisfaction. Unfortunately, there was little evidence of effective interventions to address pediatric nurse burnout. Given the prevalence and impact of burnout on a variety of important outcomes, it is imperative that nursing schools, nursing management, healthcare organizations, and nursing professional associations work to develop and test the interventions to address key attitudinal and environmental factors that are most relevant to pediatric nurses.

Burnout has been a widely studied topic of interest over the last 40 years, with significant resources devoted toward investigating its causes, impacts, and strategies for mitigation [1]. Burnout is a work outcome, defined by prolonged occupational stress in an individual that presents as emotional exhaustion, depersonalization, and diminished personal accomplishment [2].

The study of burnout in healthcare professionals is important as it has impacts at the level of the individual provider [3–5], the patient [6–9], and the organization [5, 10–12]. As nurses make up the largest group of healthcare professionals, there have been a number of studies that have explored contributing factors [13] and interventions for their burnout [14]. Pediatric nurses are a lesser-studied population, perhaps due to the relatively small number of pediatric nurses compared to general service nurses and the broader population of healthcare professionals. Burnout in pediatric nurses may be unique from adult care nurses because of the specialized nature of providing care to children who are typically seen as a vulnerable population, the high potential for empathetic engagement, and the inherent complexities in the relationships with families [15, 16]. Only one literature review could be located on the topic of pediatric nurse burnout; it mainly focused on burnout prevalence, which was found to be moderate to high [17]. Further synthesis of the literature in other domains of the topic is needed to explore factors associated with pediatric nurse burnout, the associated outcomes, and interventions.

The purpose of this scoping review is to explore what is known about pediatric nurse burnout to guide future research on this highly specialized population and, ultimately, improve both pediatric nurse and patient well-being. More specifically, the aim of this scoping review was to investigate, among pediatric nurses, (i) the prevalence and/or degree of burnout, (ii) the factors related to burnout, (iii) the outcomes of burnout, and (iv) the interventions that have been applied to prevent and/or mitigate burnout.

## Methods

### Protocol registration

This scoping review was performed according to the Preferred Reporting Items for Systematic Reviews and Meta-Analyses (PRISMA) guidelines scoping review extension [18]. The protocol was registered on Open Science Framework on 25 March 2019 and can be accessed at https://osf.io/5xrkg/.

### Information sources and search strategy

In consultation with an experienced librarian, the following electronic databases were searched on 3 November 2018 without limitation to a publication date range in order to maximize inclusion: The Cochrane Library, CINAHL, EMBASE, MEDLINE, PsycINFO, and ASSIA. All electronic database search strategies used in this review can be found in Appendix A. The term “pediatrics” was not part of the electronic database search to avoid inadvertently excluding studies that contained pediatric nurses as a non-primary subject group. The selected articles from the electronic database search were screened for inclusion of the pediatric nurse population. For the purposes of this review, the pediatric patient population is defined as newborn to age 21 as defined by the American Academy of Pediatrics, acknowledging that this age range may be slightly extended based on the country and patient needs [19].

### Eligibility criteria

All qualitative, quantitative, or mixed-methods studies published in English that examined the prevalence and/or degree of burnout in pediatric nurses using self-identification or self-report assessment tools were included. Commentaries, letters, and editorials were excluded as these are not peer-reviewed and often referred to colloquial definitions, not the clinical definition of burnout of interest in this scoping review. Dissertations were excluded, but their corresponding publications were screened for inclusion. Conference abstracts were excluded as they are often inconsistent with their corresponding full reports [20]. Systematic or scoping reviews and meta-synthesis were excluded, but references were hand-screened for suitable studies.

### Selection of sources of evidence

All citations retrieved from the databases were uploaded into Endnote with duplicates removed as per protocol [21]. The remaining citations were uploaded into Covidence for review by the research team (LB, CM, KW). Titles and abstracts were independently reviewed against the selection criteria in a blinded process by two reviewers (LB and CM). The remaining citations were then reviewed as full-text articles for inclusion against the selection criteria in a blinded process by two reviewers (LB and CM). Disagreements were resolved by a third reviewer (KW).

### Data charting process

Data were extracted from included articles and entered into a Microsoft Excel spreadsheet. Extraction was performed by one researcher (LB). The following data items were extracted: title, journal, authors, year of publication, country of publication, sample size, study aim, study design, tool used to measure burnout, burnout prevalence and scores, factors that contribute to the development of burnout in pediatric nurses, factors that prevent or mitigate burnout in pediatric nurses, the impact of burnout in pediatric nurses, and interventions for pediatric nurse burnout.

### Synthesis of results

A quantitative synthesis specific to the prevalence and degree of burnout was completed based on the included articles that reported raw scores for any of the Maslach Burnout Inventory (MBI) subscales. A mean score was calculated by hand across studies for each subscale, by totaling the raw scores and dividing by the total number of studies that included a raw score for that subscale. The resulting mean was also categorized as low, moderate, or high burnout based on published cutoff scores [22]. Other data were synthesized qualitatively to map current evidence available to address the remaining study aims. Aims ii and iii were analyzed using directed content analysis [23] following the themes outlined by Berta et al. [24], work environment, work attitudes, and work outcomes. Aim iv data was synthesized by grouping together similar interventions and descriptively summarizing the interventions that were effective in reducing burnout. Given that the overall purpose of the review was to explore the breadth of what is currently known about burnout in pediatric nurses, a quality assessment of individual studies was not conducted [18].

## Results

### Description of the search and demographics of studies included

**Fig. 1:**
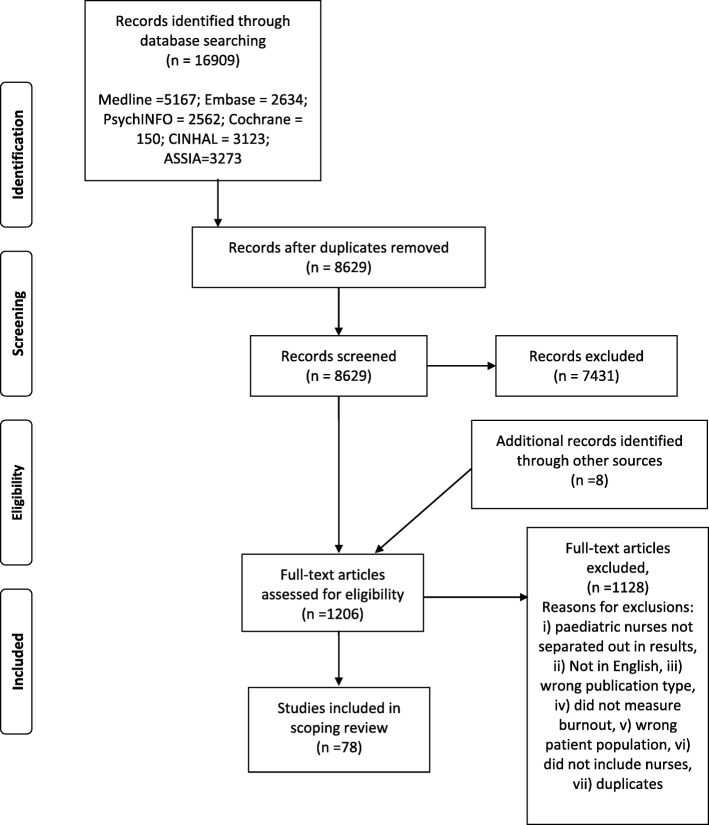
PRISMA diagram of study screening and selection

Through the initial database search, 16 909 possible papers were identified. After deduplication, 8629 titles/abstracts were screened and 1206 articles were assessed for eligibility at the level of full-text screening. After applying the inclusion and exclusion criteria, a total of 78 studies [16, 25–101] were deemed relevant and retained for analysis (Fig. 1). The characteristics of included studies are provided in Table 1. Publication dates ranged from 1981 to 2018, with the majority published between 2009 and 2017. The number of pediatric nurses who participated as either a primary or sub-sample ranged from five to 3710. The most common study design was cross-sectional (*n* = 60), with 10 studies using multi- or mixed methods, seven using an interventional design, and one each using case-control, exploratory prospective, and longitudinal designs. Only 45 of the 78 studies reviewed used exclusive samples of pediatric nurses; the remaining studies only included pediatric nurses as a subpopulation of a larger sample. The results in this review are reported for pediatric nurse samples and sub-samples only. Almost half (46%) of the included studies were conducted in the USA, followed by Canada (*n* = 7), China (*n* = 5), Turkey (*n* = 3), Brazil (*n* = 3), Taiwan (*n* = 2), Australia (*n* = 2), and Switzerland (*n* = 2), plus 18 other countries where only a study was conducted. Out of the 78 studies included, 53 (68%) used some form (either complete or abbreviated) of the MBI to measure burnout (see Table 1).

**Table 1:** Characteristics of 78 studies exploring pediatric nurse burnout included in the scoping review

Author(s), year, country	Sample	Study design	Burnout measurement tool
Adwan, 2014 [25] (USA)	120 nurses from a large academic medical center, from four pediatric patient care units and pediatric float pool nurses	Cross-sectional correlational design	MBI-HSS
Akman et al., 2016 [26] (Turkey)	165 nurses who had worked at least 1 month in pediatric clinics, surgery clinics, PICU, NICU	A descriptive, comparative, correlational cross-sectional design	MBI-HSS
Alves and Guirardello, 2016 [27] (Brazil)	267 nurses from 15 inpatient wards and three intensive care units of two pediatric hospitals	Cross-sectional correlational design	EE-HSS
Amin et al., 2015 [28] (India)	129 nurses with at least 1-year experience working in NICU	Cross-sectional design	ProQOL5
Aytekin, 2013 [29] (Turkey)	85 nurses working in two NICUs	Descriptive and correlational study (multi-methods)	MBI-HSS
Barr, 2018 [30] (Australia)	142 nurses from four NICUs	Cross-sectional cohort study	ProQOL5
Barr, 2018 [31] (Australia)	140 nurses from four NICUs	Cross-sectional survey design	ProQOL5
Berger et al., 2015 [32] (USA)	239 pediatric nurses working in a five-hospital system which included an urban pediatric tertiary care teaching hospital	Cross-sectional survey design	ProQOL5
Bilal et al., 2017 [33] (Pakistan)	113 pediatric nurses working in Punjab’s largest state-run hospital	Cross-sectional survey design	Five items adapted from the Camp scale (1994)
Bourbonnais et al., 1998 [34] (Canada)	1 891; 57 pediatric nurses employed at six acute care hospitals	Cross-sectional survey design (phase 1 of a longitudinal study)	MBI; only separated out EE
Branch and Klinkenberg, 2015 [35] (USA)	296; 179 pediatric nurses on ED, PICU, CICU, haem-onc, and cardiology units	Cross-sectional survey design	ProQOL5
Bursch et al., 2018 [36] (USA)	115 nurses working in an urban children’s hospital PICU or NICU	Cross-sectional survey design	Abbreviated MBI
Czaja et al., 2012 [37] (USA)	173 nurses on general medical, surgical and oncology wards, PICU, and ED at a tertiary care children’s hospital	Cross-sectional survey design	MBI-HSS
Davis et al., 2013 [38] (USA)	74, 15 pediatric oncology nurses from two major medical centers	Cross-sectional survey design (observational, descriptive research design)	MBI
Dos Santos Alves et al., 2017 [39] (Brazil)	267 nurses, nursing technicians, and nursing assistants on 15 in-patient units and three ICUs in two pediatric hospitals	Cross-sectional survey design	MBI: EE
Downey et al., 1995 [40] (USA)	59 NICU nurses from a single state	Multi-methods design (surveys and open-ended questions)	Adaptation of Popoff and Funkhouser ´s survey of nurses
Duxbury et al., 1984 [41] (USA)	283 nurses from 14 level 3 NICUs	Cross-sectional survey design	The Tedium Scale
Edmonds et al., 2012 [42] (Canada)	182; 88 pediatric oncology nurses from four major hospital centers	Interventional, pre/post-design	MBI
Estabrooks et al., 2011 [43] (Canada)	844 nurses from 32 units across eight pediatric hospitals	Cross-sectional survey design	MBI-GS
Faller et al., 2011 [44] (USA)	976; 117 pediatric/neonatal travel nurses employed by a large healthcare staffing company	Cross-sectional survey design	CBI
Favrod et al., 2018 [45] (Switzerland)	213; 91 NICU nurses from two university hospitals	Concurrent triangulation, mixed-methods cross-sectional study	MBI
Gallagher and Gormley, 2009 [46] (USA)	30 BMT nurses from a large pediatric medical center	Descriptive non-experimental design multi-method design (survey + an open-ended question)	MBI
Gauthier et al., 2015 [47] (USA)	45 PICU nurses at an urban pediatric academic hospital	Interventional, pre/post-design	MBI-HSS
Günüşen et al., 2018 [48] (Turkey)	117 pediatric nurses caring for children with chronic illness at an urban children’s public hospital from critical care, CV surgery, oncology, premature care, neonatal care, and burn care	Mixed-methods design (cross-sectional survey + interviews)	MBI
Habadi et al., 2018 [49] (Saudi Arabia)	182; 22 pediatric nurses working in an academic hospital including pediatric ward, NICU, and PICU	Cross-sectional survey design	MBI-HSS
Hallberg, 1994 [50] (Sweden)	11 pediatric mental health nurses from a nine-bed child psychiatric unit	Interventional mixed-methods design (supervisory sessions, surveys + open ended questions)	MBI and the Tedium Measure
Holden et al., 2011 [51] (USA)	347 nurses at two urban academic tertiary care free-standing pediatric hospitals and three inpatient units were studied at each hospital: PICU hematology and oncology transplant and general medical/surgical	Cross-sectional survey design	MBI: EE
Hsu et al., 2010 [52] (Taiwan)	121; five pediatric nurses found through the National Union of Nurses’ Associations Republic of China database	Cross-sectional survey design	Occupational Burnout Scale
Hylton Rushton et al., 2015 [53] (USA)	114; 20 pediatric nurses from two pediatric/neonatal units	Cross-sectional survey design	MBI
Jacobs et al., 2018 [54] (USA)	206; 47 nurses from six companies of the pediatric healthcare system and included employees from the medical center, primary care, home health, and other supporting departments in the system	Cross-sectional survey design	MBI and CBI
Kase et al., 2018 [55] (USA)	150; 43 pediatric palliative care nurses identified through the American Academy of Pediatrics	Cross-sectional survey design	The Compassion Fatigue and Satisfaction Self-Test for Helpers
Klein et al., 2017 [56] (Switzerland)	398; 302 nurses from nine level 3 NICUs	Cross-sectional survey design	A selection of 10 questions from the MBI covering each subscale
Koivula et al., 2000 [57] (Finland)	723; 21 pediatric nurses from two hospitals	Cross-sectional survey design	Paunonen’s instrument
Latimer et al., 2017 [58] (Canada)	51; 27 female NICU and PICU nurses at large tertiary pediatric hospital	Cross-sectional design (survey + fMRI)	Compassion Fatigue Scale
Lewiston et al., 1981 [59] (USA)	96; 38 nurses attending a workshop for health professionals who work with children with chronic illness	Cross-sectional survey design	MBI
Li et al., 2014 [60] (USA)	251 new nurses entering a residency program at an urban children’s hospital	Cross-sectional survey design	Compassion satisfaction and fatigue test
Liakopoulou et al., 2008 [61] (Greece)	113; 71 nurses from two pediatric departments	Cross-sectional, comparative, and correlational descriptive design	MBI
Lin et al., 2009 [62] (China)	249; 14 pediatric nurses working in a large public teaching hospital	Cross-sectional survey design	MBI-HSS
Lin et al., 2016 [63] (Taiwan)	144 female PICU nurses from seven regional teaching hospitals or higher-level medical institutions	Cross-sectional, correlational, survey design	Occupational Burnout Inventory
Liu et al., 2018 [64] (China)	1 761; 101 nurses from nine public tertiary hospitals in four provinces	Cross-sectional survey design	(Chinese) MBI-GS
Maytum et al., 2004 [65] (USA)	20 nurses recruited from throughout the state with extensive nursing expertise working with children with chronic conditions	Descriptive, qualitative study	Self-identification
Meadors et al., 2009 [66] (USA)	167; 23 nurses located nationwide working in PICU, NICU, or general pediatrics	Cross-sectional, correlational survey design	ProQL
Messmer et al., 2011 [67] (USA)	33 nurses with 2 years or less experience hired at a children’s hospital	Cross-sectional descriptive, correlational study	The Maslach Burnout Inventory (MBI)
Meyer et al., 2015 [16] (USA)	251 nurses entering an RN residency program at an urban children’s hospital	Longitudinal study	Compassion Fatigue Self-Test
Moody et al., 2013 [68] (USA and Israel)	47; 25 pediatric oncology nurses from two urban academic pediatric hematology/oncology programs	Interventional, mixed methods (survey + journal), pre/post-design	MBI
Morelius et al., 2013 [69] (Sweden)	47 nurses from two departments at a university hospital, a level 3 NICU, and a child and adolescent psychiatry inpatient ward	An exploratory, prospective design	CBI
Morrison Wylde et al., 2017 [70] (USA)	95 nurses entering a pediatric nurse residency program in a children’s hospital in an urban area	Interventional pre/post-design	The Compassion Fatigue Self-Test
Moussa and Mahmood, 2013 [71] (Egypt)	55 PICU nurses	Cross-sectional, descriptive, correlational design	MBI-HSS
Mudallal et al., 2017 [72] (Jordan)	407; 39 pediatric nurses, total sample from 11 hospitals	Cross-sectional, correlational design	MBI-HSS
Murphy-Oikonen et al., 2010 [73] (Canada)	14 NICU nurses at a regional hospital	Qualitative exploratory study; computer-assisted personal interview format	Self-identification
Neumann et al., 2018 [74] (USA)	914; 238 pediatric hematopoietic cell transplant nurses	Cross-sectional survey design	MBI
Nguyen et al., 2018 [75] (Vietnam)	500; 78 pediatric nurses from a general hospital, children’s hospital and obstetric hospital	Cross-sectional survey design	MBI:GS
Oehler et al., 1991 [77] (USA)	49 nurses in PICU, NICU, and intermediate care unit at a large tertiary medical center	Cross-sectional survey design	MBI
Oehler and Davidson, 1992 [76] (USA)	121 nurses working in a level 3 NICU	Cross-sectional correlational design	MBI
Ohue et al., 2011 [78] (Japan)	336; 27 pediatric nurses from three acute care hospitals	Cross-sectional survey design	MBI (revised version)
Pagel and Wittmann, 1986 [79] (USA)	74 nurses working in 13 acute care pediatric settings across three large medical centers and 10 community hospitals	Multi-methods survey design	The Tedium Measure
Paula Vega et al., 2017 [98] (Chile)	153 nurses working in pediatric oncology or PICU	Cross-sectional design	MBI
Profit et al., 2014 [80] (USA)	2 073; 1 499 RNs/NPs from 44 NICUs	Cross-sectional survey study	Four-item EE scale, based on the MBI
Richter et al., 2012 [81] (South Africa)	17 nurses working in the pediatric ward of an overburdened public hospital where intervention was rolled out	Interventional pre/post-design	MBI-HSS
Robins et al., 2009 [82] (USA)	314; 136 nurses from the Divisions of Oncology, Cardiology, Nephrology, Emergency Services, Intensive Care Units, Rehabilitation/Child Development, Psychology, Social Work, and Anesthesiology. General medical nurses who provided care on the medical/surgical and critical care floors but did not belong to a specific pediatric division were also recruited.	Cross-sectional design	Compassion Satisfaction and Fatigue Test
Rochefort and Clarke, 2010 [83] (Canada)	339 NICU nurses working in nine large urban, publicly-administered teaching hospitals	Cross-sectional correlational survey design	MBI: EE
Rodrigues et al., 2018 [85] (USA)	32 and 41; study 1: 32 nurses working in a 44-bed pediatric inpatient care unit at a large urban hospital; study 2: participants included 41 nurses working in general pediatric inpatient units at two large urban children’s hospitals (27 from site 1 and 14 from site 2)	Mixed methods (focus groups + surveys)	Study 1: self-report; study 2: MBI: EE, DP
Rodrigues et al., 2018 [84] (USA)	33 nurses working on a 44-bed pediatric inpatient care unit at a large urban hospital	Interventional, post-intervention, repeated measures design	MBI: EE, DP
Rodriguez-Rey et al., 2018 [86] (Spain)	487; 281 nurses from PICU and general pediatrics at nine hospitals	Cross-sectional survey design	MBI
Roney and Acri, 2018 [87] (USA)	318 nurses—current members of the Society of Pediatric Nurses (SPN)	Cross-sectional survey design	ProQOL
Sekol and Kim, 2014 [88] (USA)	240 nurses working in surgical, medical, critical care, and hematology/oncology units of a children’s hospital	Cross-sectional survey design	ProQOL5
Skorobogatova et al., 2017 [89] (Lithuania)	94 nurses working in NICUs of two tertiary care perinatology centers	Cross-sectional survey design	MBI-HSS
Soroush et al., 2016 [90] (Iran)	86 NICU nurses from all educational hospitals in the region	Cross-sectional, descriptive, survey design	MBI
Squires et al., 2013 [91] (Canada)	735 nurses from 15 children’s hospitals	Cross-sectional survey design	MBI-GS
Stimpfel et al., 2013 [92] (USA)	3 710 NICU, PICU, newborn nursery, and general pediatrics nurses from 342 hospitals	Cross-sectional survey design	MBI-HSS
Sun et al., 2017 [93] (China)	602; 51 pediatric nurses from three hospitals	Cross-sectional survey design	MBI-HSS
Sun et al., 1996 [94] (China)	1 100; 277 pediatric nurses from multiple medical centers	Cross-sectional survey design	MBI
Tawfik et al., 2017 [95] (USA)	1 934; 1 374 NICU nurses from 44 NICUs in the same state	Cross-sectional survey design	MBI
Tawfik et al., 2017 [96] (USA)	2 073; 1 464 NICU nurses from 44 NICUs in the same state	Cross-sectional design	MBI
Vicentic et al., 2016 [97] (Serbia)	60; 30 nurses/technicians of children with CP; control group of 30 pediatric nurses/technicians, as caregivers of normally developing, non-palsy children	Case-control study	MBI-HSS
Watson and Feld, 1996 [99] (New Zealand)	14 pediatric ward nurses	Multi-methods design (surveys and open-ended questions)	MBI
Yao et al., 2018 [100] (China)	860; 44 pediatric nurses	Cross-sectional design	MBI-GS
Zanatta and Lucca, 2015 [101] (Brazil)	188; 57 pediatric nurses at a single institution	Cross-sectional design	MBI-HSS

Total sample size is provided, in addition to sample size of pediatric nurses, if pediatric nurses were a subgroup of a larger sample

*MBI* Maslach Burnout Inventory; *MBI-GS* Maslach Burnout Inventory-General Survey; *MBI-HSS* Maslach Burnout Inventory-Human Services Survey; *EE* Emotional Exhaustion, a subscale of the Maslach Burnout Inventory; *DP* Depersonalization, a subscale of the Maslach Burnout Inventory; *CBI* Copenhagen Burnout Inventory; *ProQOL* Professional Quality of Life Scale; *ProQOL5* Professional Quality of Life Scale, version 5; *ED* emergency department; *PICU* pediatric intensive care unit; *NICU* neonatal intensive care unit; *CICU* cardiovascular intensive care unit; *Haem-onc* hematology-oncology; *BMT* bone marrow transplant; *CP* cerebral palsy

### Burnout prevalence and scores

Although all of the included studies measured burnout using a self-report assessment tool or binary self-identification, only 65 reported burnout scores for a sample of pediatric nurses (Table 2). In total, 53 studies used the MBI, 34 reported on the *Emotional Exhaustion* subscale with 24 reporting raw scores [25, 27, 29, 39, 42, 45, 48, 53, 61, 62, 72, 75, 77, 78, 84–86, 89, 90, 94, 97, 99, 100], and 16 reporting proportions and/or severity of those with scores indicating emotional exhaustion (e.g., low, moderate, high). The mean of the reported raw Emotional Exhaustion scores was 22.45 (SD = 6.54) which indicates moderate burnout [22]. Out of the 14 studies reporting the proportion of respondents with scores indicating *high* emotional exhaustion, the mean proportion was 38.7%. The mean of the reported raw *Depersonalization* scores [25, 29, 42, 45, 47, 48, 53, 61, 62, 72, 75, 77, 78, 84–86, 89, 90, 94, 97, 99, 100] was 6.95 (SD = 3.38) which indicates moderate burnout [22]. The mean of the reported raw *Personal Accomplishment* scores [25, 29, 42, 45, 47, 48, 53, 61, 62, 72, 75, 77, 78, 86, 89, 90, 94, 97, 99, 100] was 29.15 (SD = 11.48) which indicates high personal accomplishment [22]. The individual scores from the MBI and other measurement tools are reported for each study in Table 2.

**Table 2:** Pediatric nurse burnout scores by tool

Maslach Burnout Inventory						
Author(s)	Number of pediatric nurses	Adaption to the tool	Additional/alternative results	Personal Accomplishment subscale	Depersonalization Subscale	Emotional Exhaustion Subscale
Adwan [25]	120	No		**PA:** min-max, 13–48, avg. 38, SD = 5.7 (moderate)	**DP:** 0–19, avg. 5.8, SD = 4.7 (low)	**EE:** 5–45, avg. 21, SD = 8.58 (moderate)
Akman et al. [26]	165	No		**PA**: high	**DP**: low	**EE**: low
Alves and Guirardello [27]	267	Yes	EE only			**EE:** min-max, 9–39, avg. = 21.5, SD = 6 (moderate)
Aytekin et al. [29]	85	No		**PA:** min-max, 1–22, avg. = 11.43, SD = 4.63	**DP:** min-max, 0–13, avg. = 3.87, SD = 2.77	**EE:** min-max, 3–27, avg. = 14.9, SD = 5.53
Bourbonnais et al. [34]	57	Yes	Separated out EE only			**High EE:** 20 (35%) Crude Prevalence Ratio: 1.04
Czaja et al. [37]	173	No		46% low **PA**	38% had high **DP**	45% had **EE**
Davis et al. [38]	15	No		**PA:** 6.67% low, 53.3% mod, 40% high		
Dos Santos Alves et al. [39]	267	No				Moderate **EE:** mean 21.5 (SD ± 6); high **EE:** 27%
Edmonds et al. [42]	88	No		Pre-intervention: **PA:** mean 35.9 (6.4), 30.7% low **PA**	Pre-intervention: **DP**: mean 6.1(4.8), 25% high	Pre-intervention: **EE:** mean 22.3 (9.7), 24.1% high Post-intervention: **EE** change: mean 2.2 (6.3) Wilcoxon signed-rank 0.007 7-month post-intervention: **EE** change: mean 2.3 (8.6) Wilcoxon signed-rank 0.004
Favrod et al. [45]	91	No		**PA:** mean 31.6 (5.5), high = 3.6%, mod. = 35.7%, low = 60.7%	**DP**: mean 4.8 (4.1), low = 64.3%, mod. = 29.8%, high = 6.0%	**EE**: mean = 23(9.9), low = 31%, mod = 47.6%, high = 21.4%
Gallagher and Gormley [46]	30	No		**PA:** low = 16.7%, mod. = 33.3%, high = 50%	**DP:** low = 63.3%, mod. = 33.3%, high = 3.3%	**EE:** low = 26.7%, mod. = 46.7%, High = 26.7%
Gauthier et al. [47]	45	No	Pre/post-intervention trends: significant quadratic trajectory of PA in which PA increased at time 2 and decreased at time 3 [F(1, 35) 5.43, *P* = 0.03], with no significant trends for EE and DP	Baseline: high **PA** mean 47.29 (7.43)	Baseline: High **DP**, mean = 13.33 (5.84)	Baseline: High **EE**, mean = 31.39 (9.85)
Günüşen et al. [48]	117	No		**PA:** 19.20 (4.18)	**DP**: 6.14 (2.98),	**EE:** 18.85 (6.21)
Habadi et al. [49]	22	No	**Burnout** = 0.55%	Low **PA**: 36.4%	High **DP**: 22.7%	High **EE**: 59%
Hylton et al. [53]	20	No		**EE:** 39.9 (7.1)	**DP**: 12.5 (6.2)	**EE:** 33 (13.8)
Jacobs et al. [54]	47	Yes—used in conjunction with Copenhagen Burnout Inventory	Analysis of subgroup differences between nurses and non-nurses indicated a trend toward nurses reporting higher work-related burnout (39.92 vs. 35.86, *P* < .067) and client-related burnout (23.88 vs. 18.96, *P* < .061).			
Klein et al. [56]	302	Yes—selection of 10 questions of the Maslach Burnout Inventory: MBI covering each subscale	“I have accomplished many worthwhile things in this job” = 4.32 (4.13–4.50); “I feel very energetic” = 4.11 (3.93–4.28); “In my work, I deal with emotional problems very calmly” = 3.07 (2.83–3.31); “I feel fatigued when I get up in the morning and have to face another day on the job” = 1.91 (1.74–2.08); “I feel emotionally drained from my work” = 1.29 (1.16–1.41); “I feel burned out from my work” = 1.26 (1.13–1.40); “I feel frustrated by my job” = 1.26 (1.14–1.38); “I worry that this job is hardening me emotionally” = 0.89 (0.74–1.04); “I do not really care what happens to some patients” = 0.41 (0.30–0.53); “I feel I treat some patients as if they were impersonal objects” = 0.24 (0.16–0.32)			
Lewiston et al. [59]	38	No	9 RNs had burnout greater than the mean (high burnout) and 11 had burnout below the mean (low burnout)			
Liakopoulou et al. [61]	71	No		**PA** mean = 37.8 (5.8)	**DP** mean = 5.2 (4.9)	**EE** mean = 27.5 (9.5)
Lin et al. [62]	14	No		**PA** mean = 34.5 (7.1) (moderate)	**DP** mean = 3.6 (4.0) (low)	**EE** mean = 24.2 (15.6) (moderate)
Liu et al. [64]	101	No	mean = 7.09 (3.23) (lower job burnout)			
Moussa and Mahmood [71]	55	No		**PA:** high level burnout 14.5%, low level burnout 85.5%	**DP:** high level burnout 74.5%, low level burnout 25.5%	**EE:** high level burnout 56.4%, low level burnout 43.6%;
Mudallal et al. [72]	39	No		**PA:** mean = 31.23 (12.14)	**DP**: mean = 12.74 (4.89)	**EE:** mean = 28.33 (12.63)
Nguyen et al. [75]	78	No	16.7% burned out, 14.1% exhausted, 3.8% depressed, 65.4% healthy	**PA** = 4.06 (0.61)	**DP** mean = 2.86 (0.73)	**EE** mean = 3.12 (0.95)
Oehler and Davidson [76]	121	No	Overall, scores represent moderate EE, DP, and high lack of PA	low **PA** mean = 36.6, mod **PA** mean = 32.8, high **PA** mean = 30.6	low **DP** mean = 2.7, mod **DP** mean = 7.2, high **DP** mean = 11.9	Low **EE** mean = 12.2, mod **EE** mean = 24.2, high **EE** mean = 32.7
Oehler et al. [77]	49	No		**PA** mean = 32.1 (6.9) (high)	**DP** mean = 6.9 (4.9) (moderate)	**EE** mean = 24.1 (10.1) (moderate)
Ohue et al. [78]	27	Yes—revised version of MBI		**PA** mean = 14.44 (4.26)	**DP** mean = 12.44 (4.21)	**EE** mean = 17.44 (3.61)
Paula Vega et al. [98]	153	No		79.7% (*n* = 122) showed medium to low level of **PA**, and it was more evident in oncology professionals, as 41% of them showed low **PA**.	16% (*n* = 11) had a high level of **DP**.	48.4% (*n* = 74) of participants had a medium or high level of **EE**.
Profit et al. [80]	1 499	Yes—four-item Emotional Exhaustion scale, based on the MBI	Nurses reporting burnout mean = 26.9%, SD = 11.4, *P* = 0.0004 (based on the authors determined cutoff)			
Richter et al. [81]	17	No		Nurses in the pilot study experienced average levels of **PA** during the pilot phase of the study. No changes across the pre/post-intervention phases.	Nurses in the pilot study experienced low levels of **DP** and no changes across the pre/post-intervention phases.	Nurses in the pilot study experienced high levels of **EE** and no changes across the pre/post-intervention phases.
Rochefort and Clarke [83]	339	Yes—only EE subscale				35.7% had **EE** scores higher than the published norms for medical personnel.
Rodrigues et al. [85]	73	Yes—only EE and DP subscales			**DP** mean = 6.24 (4.67) (20% have high DP)	**EE** mean = 24.39 (11.68) (46% have high **EE**)
Rodrigues et al. [84]	33	Yes—only EE and DP subscales			**DP** pre: 11.34 (4.66), **DP** post: 9.25 (3.23) 63% reported high **DP** at baseline, and 3 months after the intervention, there were still 34% reporting high **DP**.	**EE** pre: 32.38 (11.29), **EE** post: 29.47 (10.52) 73% of our nurses reported high **EE** at baseline, and 3 months after the intervention, there were still 47% of our nurses reporting high **EE**.
Rodriguez-Rey et al. [86]	281	No		**PA** mean = 37.74 (6.88)	**DP** mean = 6.41 (4.56)	**EE** mean = 21.74 (9.17)
Skorobogatova et al. [89]	94	No		**PA** mean = 29.1 (10.12); low levels of self-esteem and self-efficacy and achievement (as components of professional burnout) were found in 61.7%, and moderate levels were found in 23.4% of respondents	**DP** mean = 3.8 (4.75); moderate levels of **DP** in 9.6% and high levels in 12.8% of nurses	**EE** mean = 14.4 (7.91); overall, moderate **EE** was common in 41.5% and high in 9.6% of neonatal nurses.
Soroush et al. [90]	86	No	Burnout mean = 46.2 (12.5) (mod)	**PA** mean = 22.6 (5.4 (strong lack of **PA**))	**DP** mean = 2.6 (31) (weak)	**EE** mean = 21.28 (8.1 (mod))
Stimpfel et al. [92]	3 710	No	Nurses who work 8 h shifts: 25% burned out; nurses who work 12 h shifts: 24% burned out; nurses who work > 13 h shifts: 46% burned out			
Sun et al. [93]	51	No	14 (27.5%) were positive for burnout, and 37 (72.5%) were negative for burnout.			
Sun et al. [94]	277	No		**PA** mean = 33.88 (2.77)	**DP** mean = 10.97 (1.67)	**EE** mean = 21.45 (2.92)
Vicentic et al. [97]	60	No		*Nurses/techs for children with CP:* **PA** mean = 36.40 (6.473) (30% low risk, 40% mod risk, 30% high risk) *Nurses/tech for children without CP:* **PA** mean = 36.37 (11.180) (47% low risk, 30% mod risk, 23% high risk)	*Nurses/techs for children with CP:* **DP** mean = 4.31 (5.594) (87% low risk, 13% high risk) *Nurses/tech for children without CP:* **DP** mean = 4.47 (4.584) (83% low risk, 17% high risk)	*Nurses/techs for children with CP:* **EE** mean = 25.67 (15.043) (33% low risk, 17% mod risk, 50% high risk *Nurses/tech for children without CP:* **EE** mean = 17.57 (10.153)(53% low risk, 30% mod risk, 17% high risk)
Watson and Feld [99]	14	No		**PA** mean = 34.93 (4.53)	**DP** mean = 4.86 (4.41)	**EE** mean = 22.27 (10.31)
Yao et al. [100]	44	No		**PA** mean = 11.0 (9.7)	**DP** mean = 7.0 (6.1)	**EE** mean = 32.8 (15.5)
Zanatta and Lucca [101]	57	No		**PA**: high 24.6%, mod 52.6%, low 22.8%	**DP**: high 29.8%, mod 43.9%, low 26.3%	**EE**: high 24.6%, mod 49.1%, low 26.3%
Professional Quality of Life Scale						
Author(s)		Number of pediatric nurses		Results		
Amin et al. [28]		129		**Low:** 26.4% (34)	**Moderate:** 50.4% (65)	**High:** 23.3% (30)
Barr [30]		142		mean = 2.4, SD = .52		
Berger et al. [32]		239		**Low:** 23% (55)	**Moderate:** 47.7% (114)	**High:** 29.3 (70)
Branch and Klinkenberg [35]		179		Mean = 49.7 (9.6)		
Meadors et al. [66]		23		Mean = 14.82 (4.33)		
Roney and Acri [87]		318		The 25th percentile ranking for the Burnout subscale of the Professional Quality of Life (ProQOL) measure was 43.45, the 50th percentile ranking was 49.22, and the 75th percentile was 56.92.		
				The average score on the burnout subscale is 50, which is higher than the 50th percentile ranking in this current study (slightly lower than average levels of burnout).		
Sekol and Kim [88]		240		Surgical unit burnout = 24.5 (5.1); medical unit burnout = 22.8 (4.7); critical care unit burnout = 23.0 (4.7); hematology/oncology unit burnout = 20.2 (3.9)		
Compassion Fatigue Self-Test						
Author(s)		Number of pediatric nurses		Results		
Kase et al. [55]		43		4.7% prevalence		
Latimer et al. [58]		27		Mean = 23(10.8) (significantly higher than pediatric allied health providers)		
Li et al. [60]		251		Mean = 24.01 (SD = 11.67)		
Meyer et al. [16]		251		Mean = 24.01 (SD = 11.67)		
Morrison Wylde et al. [70]		95		Score after smartphone-delivered mindfulness: 22.37 (11.90)		
				Score after traditional delivered mindfulness: 26.14 (11.25)		
Robins et al. [82]		136		Mean = 27.8 (10.6)—extremely low risk		
The Tedium Measure						
Author(s)		Number of pediatric nurses		Results		
Pagel and Wittmann [79]		74		The burnout scale mean for all subjects was 3.5855 with a standard deviation of .701. The scores ranged from 1.7142 to 5.4766 (signifies evidence of burnout).		
The Copenhagen Burnout Inventory						
Author(s)		Number of pediatric nurses		Results		
Faller et al. [44]		117		Half had high work-related burnout, and half had low work-related burnout.		
Jacobs et al. [54]		47		Analysis of subgroup differences between nurses and non-nurses indicated a trend toward nurses reporting higher work-related burnout (39.92 vs. 35.86, *P* < .067) and patient-related burnout (23.88 vs. 18.96, *P* < .061). *Conducted with MBI		
Morelius et al. [69]		47		**NICU nurses:** CBI, personal related burnout: 32.4 (13.8); work-related burnout: 24.3 (9.9); client-related burnout: 13.3 (9.1)		
				**Child and adolescent psychiatry nurses:** personal-related burnout: 32.4 (14.5); work-related burnout: 28.1 (16.3); client-related burnout: 22.9 (15.7)		
Occupational Burnout Inventory						
Author(s)		Number of pediatric nurses		Results		
Hsu et al. [52]		5		Mean = 60.00 (SD = 11.75) (scale is out of 90, higher = greater burnout)		
Jacobs et al. [54]		47		Analysis of subgroup differences between nurses and non-nurses indicated a trend toward nurses reporting higher work-related burnout (39.92 vs. 35.86, *P* < .067) and patient-related burnout (23.88 vs. 18.96, *P* < .061).		
Paunonen’s instrument						
Author(s)		Number of pediatric nurses		Results		
Koivula et al. [57]		21		Mean = 6.15, SD = (0.71) (mild)		
Popoff and Funkhouser’s survey of nurses (adapted version)						
Author(s)		Number of pediatric nurses		Personal Accomplishment subscale		
Downey et al. [40]		59		**PA** mean = 1.0 (0.8)		

*MBI* Maslach Burnout Inventory; *EE* Emotional Exhaustion, a subscale of the Maslach Burnout Inventory; *DP* Depersonalization, a subscale of the Maslach Burnout Inventory; *PA* Personal Accomplishment, a subscale of the Maslach Burnout Inventory; *CP* cerebral palsy; *ProQOL* Professional Quality of Life Scale; *CBI* Copenhagen Burnout Inventory; *SD* standard deviation, *NICU* neonatal intensive care; *Mod* moderate

### Factors related to burnout

Of the included studies, 47 (60%) addressed factors associated with pediatric nurse burnout (Table 3). Factors related to pediatric nurse burnout were classified into the following categories: nurse demographics, work environment, work attitudes, work outcomes, and burnout interventions.

**Table 3:** Factors associated with pediatric nurse burnout

Author(s)	Number of pediatric nurses	Factors associated with burnout
Akman et al. [26]	165	Higher **EE** scores associated with working in emergency and surgery, moderate in internal med, PICU, NICU
		Low **DP scores** associated with working in PICU, moderate in all other units
		High **PA**, high all units.
		Lower level of burnout associated with a high level of job satisfaction, being married, increased age, and decreased number of assigned patients
Amin et al. [28]	129	Higher burnout associated with greater perceived stress
Aytekin et al. [29]	85	Higher **EE** scores associated with being unhappy with their work environment
		Lower **EE** associated with working at management level in NICU over other NICU nurses
		Lower **PA** scores associated with longer years worked in the NICU
Barr [30]	142	Core-self evaluations explained 33% variance in burnout
		Degree of agreeableness, neuroticism, extraversion, and positive affect contributed to variances in burnout
		Positive affect mediated the effect of core self-evaluations on burnout
Barr [31]	140	Higher burnout associated with high neuroticism and low agreeableness and work stress, controlled for personality traits
		Work stress mediated the effect of neuroticism and extraversion on burnout
Berger et al. [32]	239	Higher burnout and lower compassion satisfaction associated with nurses under 40 years of age, with 6–10 years of experience and/or working in a medical-surgical unit
Bilal et al. [33]	113	Higher burnout associated with an organizational structure with rules and relations and being a supervisor
		Lower burnout and burnout prevention associated with participation in decision-making, instrumental communication, and promotional opportunities
Branch and Klinkenberg [35]	179	Higher burnout associated with nursing in PICU over other units
Bursch et al. [36]	115	Higher **EE** associated with nurses working most frequently in the PICU relative to those working most frequently in the NICU, those who found communication with nurses more stressful, and having a lack of necessary nursing supplies
		Lower **EE** associated with being married or in a domestic partnership relative to respondents who were unmarried and not in a domestic partnership, identifying as Asian/Pacific Islander relative to respondents identifying as White
		Higher **DP** associated with respondents working day shifts relative to those working the night shift or a mix of day and night shifts, nurses working most frequently in the PICU relative to those working most frequently in the NICU, greater endorsement of stress related to communication among nurses, the experience level of colleagues, staffing, and stress associated with the patient population
		Lower **DP** associated with respondents working in the NICU relative to those working frequently in the PICU and respondents who reported being married or in a domestic partnership
		Higher **PA** reported in individuals identifying as White and individuals identifying as Asian/Pacific Islander relative to individuals identifying as others
		Lower **PA** found in nurses who found their own lack of knowledge, skills and/or confidence in themselves stressful and respondents identifying as being of another ethnicity/race relative to respondents identifying as White
Czaja et al. [37]	173	Lack of burnout or PTSD associated with nurses who generally felt more positively about their work environment, with more confidence in their physician and nurse collogues as well as feeling a part of a team
Davis et al. [38]	15	Higher **PA** associated with working in adult oncology over pediatric oncology nurses
Dos Santos Alves et al. [39]	267	Lower burnout associated with nurses with a perception of having greater autonomy, greater control, good relationships at work, and organizational support, and are more satisfied with the work and the safety climate is assessed as more positive
Duxbury et al. [41]	283	Higher burnout found in staff nurses who have a head nurse with a leadership style of high structure and low consideration
Estabrooks et al. [43]	844	Higher **EE** associated with lower job satisfaction
Favrod et al. [45]	91	Similar burnout levels in NICU nurses and midwives
		NICU nurses more likely to reach the severe threshold of the three subscales of burnout than midwives
		NICU nurses reported more traumatic stressors in their working environment
Gallagher and Gormley [46]	30	Higher **EE** associated with higher **DP** and low **PA**, **EE** still present despite nurses reporting support systems were in place and felt supported
		Lower **EE** associated with increased years as a BMT nurse
		Lower **DP** associated with increased years as a BMT nurse
		Higher **PA** associated with increased years as a BMT nurse
Gauthier et al. [47]	45	Lower **EE** associated with self-compassion at time 1 and time 2, but not at time 3
		Lower **DP** associated with elf-compassion at time 1 and time 2, but not at time 3
		Higher **PA** associated with self-compassion at all three time points
		All subscales of burnout were correlated with job satisfaction at time 1, but not at time 2 and time 3
		Lower burnout associated with more years of experience, job satisfaction had a significant positive correlation with stress and burnout only at time 1
Holden et al. [51]	347	Higher burnout associated with unit-level staffing, task-level external mental workload, and job dissatisfaction
		Burnout and job dissatisfaction were not significantly associated with the likelihood of medication error
Klein et al. [56]	302	Nurses rated lack of regular staff meetings, dissatisfaction with the quality of the decision-making process, and providing futile treatment as significantly more stressful than physicians did
Koivula et al. [57]	21	Higher burnout found in nurses with lower education level relative to those with higher education level
Latimer et al. [58]	27	Higher burnout associated with nurses with less experience
Lewiston et al. [59]	38	Higher **EE** associated with cystic fibrosis caregivers compared to controls
		Higher **DP** associated with cystic fibrosis caregivers compared to controls
		Equal **PA** from the job in cystic fibrosis caregivers and the control group
Lin et al. [63]	144	Higher burnout associated with higher work stress (after controlling for the demographics) and depression
		Occupational burnout had a mediating effect on the relationship between work stress and depression levels
Maytum et al. [65]	20	**Factors associated with triggering burnout:** seeing too many painful procedures done to children, seeing too much sadness, seeing too much death, angry, yelling families, and non-compliant patients/families
		Systems triggers: unreasonable policies, staffing shortages, insurance frustrations, paperwork, need to justify their position, and general healthcare system dysfunction
		Role-specific triggers: lack of support, feeling you are on your own, less respondents cited unclear expectations, change in role and lack of challenge
		Work overload: excessive demands of work
		Personal triggers becoming overly involved or crossing professional boundaries
		**Factors associated with coping with burnout:** short-term—self-care (exercise, meditation, journaling), fun/humor, non-work relationships; long-term personal coping strategies—developing a personal philosophy and faith and engaging in self-analysis
		Short-term work-related coping strategies: developing supportive and honest professional relationships, need for their work to be congruent with their professional philosophy and interest
Messmer et al. [67]	33	Higher burnout associated with lower satisfaction and position
		Lower burnout associated with nurses who would recommend their career to others relative to those who would recommend their career with reservation
Meyer et al. [16]	251	Higher burnout predicated by current stress exposure after controlling for pre-existing stress exposure
Morrison Wylde et al. [70]	95	Lower burnout associated with “acting with awareness” at time 2
Moussa and Mahmood [71]	55	Higher **EE** associated with lack of access to work information
		Lower **EE** associated with nurses increased age, length of professional experience, and experience on the unit
		Lower **DP** associated with nurses increased age, length of professional experience, and experience on the unit
		Higher **PA** associated with nurses increased age, length of professional experience, and experience on the unit
Murphy-Oikonen et al. [73]	14	Higher burnout and frustration when caring for infants with neonatal abstinence syndrome
Neumann et al. [74]	238	Lower **EE** associated with caring for both pediatric and adult patients had lower relative to those who just cared for adults only
Oehler and Davidson [76]	121	Higher and more frequent burnout found in acute pediatric nurses relative to non-acute pediatric nurses
		Higher burnout associated with increased job stress, workload, conflict with physicians, and uncertainty regarding treatment
Oehler et al. [77]	49	Higher **EE** predicted by job stress, trait anxiety, and experience on the current unit and explained 55% of the variance
		Higher **DP** predicted by job stress and total work experience
		Lower **PA** predicted by level of supervisor support and state anxiety
Ohue et al. [78]	27	Higher **PA** found in nurses in the pediatrics and outpatient departments relative to those of the nurses in the obstetrics and gynecology departments
Pagel and Wittmann [79]	74	Higher burnout related to higher reporting of the variable “percentage of children on a unit with social of behavioral problems”
Rochefort and Clarke [83]	339	Lower **EE** associated with higher ratings of nurse staffing and resource adequacy
Rodrigues et al. [85]	73	Higher **EE** associated with greater time on the unit (moderate effect), nurses concern that current standards of care inhibit optimal pain management, negative views of the hospital environment (large effect), barriers to optimal pain management (moderate effect), lower self-efficacy (moderate effect), and moral distress (moderate effect)
		Burnout associated with expressions of exhaustion, frustration, overburden of their workload, and the hopelessness in working with chronically ill pediatric patients, issues about self-efficacy regarding patient outcomes
Sekol and Kim [88]	240	Higher burnout found in those with 5–9 years of experience working on the surgical unit
		Lower burnout associated with working on the hematolgy/oncology unit, nursing experience of > 20 years, and all levels of experience if working on the hematolgy/oncology unit
Soroush et al. [90]	86	Higher burnout associated with low clinical competency
Squires et al. [91]	735	Higher **DP** associated with lower application of research information in the work context
Stimpfel et al. [92]	3 710	Higher burnout associated with nurses who worked the longest shifts relative to those working shorter, 8-h shifts
Sun et al. [94]	277	Higher burnout in nurses who worked in obstetrics and gynecology units relative to nurses who worked in the surgery and pediatric units, in that order
Tawfik et al. [95]	1 374	Higher burnout associated with an average number of daily admissions of the NICU
Tawfik et al. [96]	1 464	Higher burnout in understaffed units
Vicentic et al. [97]	60	Higher **EE** associated with higher anxiety and depression variables and higher risk of **EE** for those who care for children with CP than those who care for children without CP
Zanatta and Lucca [101]	57	Higher **EE** associated with being married and having health problems related to work

*EE* Emotional Exhaustion, a subscale of the Maslach Burnout Inventory; *DP* Depersonalization, a subscale of the Maslach Burnout Inventory; *PA* Personal Accomplishment, a subscale of the Maslach Burnout Inventory; *NICU* neonatal intensive care unit; *PICU* pediatric intensive care unit; *STS* secondary traumatic stress; *PTSD* post-traumatic stress disorder; *CP* cerebral palsy

#### Nurse personal factors

Burnout was found to be inversely associated with age; higher burnout was also associated with low/moderate level of experience (5–10 years) [26, 32, 46, 47, 58, 71, 85, 88]. A lack of university-level education or lower self-reported levels of clinical competency were also associated with higher levels of burnout [57, 90]. Being in a nursing supervisory position had ambiguous results on impact on burnout; in some studies, holding supervisory positions correlated with higher reports of burnout while in others, the opposite effects were found [29, 33]. Nurses identifying as not being White or Asian/Pacific Islander ethnicity/race scored significantly lower on the MBI subscale of *Personal Accomplishment* than respondents identifying as White and Asian/Pacific Islander, and Asian/Pacific Islanders scored lower on *Emotional Exhaustion* than those identifying as White [36]. High neuroticism and low agreeableness [31] were both associated with higher burnout. Finally, being married had mixed results on impact on burnout, whereas in some studies, being married correlated negatively with burnout, and in others, it correlated positively [26, 36, 101].

### Work environment

The work environment is defined by the conditions in which nurses work; it influences work attitudes and, in turn, work outcomes [103]. Burnout was found to be high in certain high-acuity pediatric units including emergency, medical/surgical, surgery, pediatric intensive care unit (PICU), and neonatal intensive care unit (NICU) [26, 32, 35, 36, 76, 88]. Davis et al. [38] found that adult oncology nurses had higher personal accomplishment than pediatric oncology nurses while Neumann et al. [74] found nurses who care for both pediatric and adult patients had lower emotional exhaustion than those who cared for adult patents only. Conversely, Sun et al. [94] found that nurses who worked in adult obstetrics and gynecology units had more burnout than nurses who worked in pediatric units; however, Ohue et al. [78] reported the inverse. Working in hematology/oncology [88] and unit-level factors such as workload [65, 85], number of assigned patients [26], increased number of admissions, understaffing, and shifts > 8 h were associated with increased burnout [51, 92, 95, 96]. Aytekin et al. [29] found working longer years in the NICU was associated with lower levels of personal accomplishment. Brusch et al. [36] found that nurses working exclusively day shifts had higher levels of depersonalization than those working night shifts or a mix of days and nights. Favrod et al. [45] found NICU nurses reported more traumatic stressors in their working environment.

Pediatric nursing workplaces with a strict structure of rules and regulations [33] or nurse leaders who valued structure over staff considerations [41] were found to have nurses with higher burnout. Nurses who had higher perceived organizational support had lower burnout [39]. Higher burnout was generally associated with systems issues such as unreasonable policies, staffing shortages, insurance frustrations, high volumes of paperwork [65], lack of nursing supplies [36], and lack of regular staff meetings [56]. The relationship between resources and facets of burnout was mixed: Rochefort and Clarke [83] found a negative relationship between nurses’ emotional exhaustion and their rating of the adequacy of the resources available to them, while Gallagher and Gormley [46] found that even nurses who reported that support systems were in place and felt supported still were emotionally exhausted. The lack of access to work information and research information was consistently associated with higher levels of burnout [71, 91], and lower burnout was associated with increased communication [33, 36] and better work relationships [39].

Factors impacting increased pediatric nurse burnout were related to the role of the nurse in patient care activities such as decision-making/uncertainty around treatment [33, 39, 56, 76], lack of role clarity, and unclear plan of care [65]. Other factors associated with the development of burnout were related to exposure to suffering, pain, sadness, and death [65]; hopelessness [85]; providing futile care [56]; and overall moral distress [85].

Higher levels of burnout were found in nurses who cared for specific pediatric patient populations such as caring for children with cerebral palsy [97], children with cystic fibrosis [59], and babies with neonatal abstinence syndrome [73]. Another patient factor related to higher burnout involved behavioral issues from patients/families [65, 79].

### Work attitudes

Work attitudes are factors that impact the positive or negative perceptions of one’s work environment [104]. Low self-compassion and low mindfulness [47] were associated with higher burnout. Co-occurring conditions with burnout such as depression [37, 63, 97], anxiety [97], and somatic work-related health problems [101] were correlated with greater burnout whereas positive psychosocial factors and coping strategies such as positive affect [30], acting with awareness [70], self-care, humor, reflection, non-work relationships, and a personal philosophy related to work were found to be associated with lower burnout [65].

Nurses’ perceived work stress was positively associated with burnout in several studies [16, 28, 63, 76, 77]. Meyer et al. [16] found that current stress exposure significantly predicted higher levels of burnout after controlling for pre-existing stress exposure, and Holden et al. [51] found that burnout was positively associated with mental workload. Oehler and Davidson [76] found perceived workload made a significant contribution to feelings of burnout. Job satisfaction was also found to be negatively associated with burnout [26, 29, 39, 43, 47, 51, 67].

### Work outcomes

Work outcomes refer to occupational performance factors that are influenced by work attitudes and the work environment [24]. Nine studies examined work outcomes associated with burnout including nurse retention, nurse well-being, patient safety, and patient-family satisfaction (Table 4). An increase in burnout was associated with nurses considering a career change [37], decreased quality of life [29], tiredness [89], and feeling negatively toward their teammates and the impact of their work [37]. Work-associated compassion fatigue [16, 66], secondary traumatic stress [48, 58], and post-traumatic stress disorder (PTSD) [37, 60, 70] were all found to be associated with pediatric nurse burnout. However, Li et al. [60] report that high group cohesion may prevent pediatric nurses from developing burnout from PTSD by protecting nurses from the impacts of negative outcomes. Nurse burnout was found to be negatively associated with the safety climate of the hospital in which they work [27, 39] and positively associated with higher infection rates when nurses were feeling overworked [96]. Moussa and Mahmood [71] found that as nurses’ personal accomplishment increased, so did patients’ mothers’ satisfaction with meeting their child’s care needs in the hospital.

**Table 4:** Burnout’s relationship with other work outcomes in pediatric nurses

Author(s)	Number of pediatric nurses	Burnout outcomes
Alves and Guirardello [27]	267	Higher **EE** associated with the outcome of a worse patient safety climate
Aytekin et al. [29]	85	Higher burnout associated with the outcome of decreased quality of life in the nurse
Czaja et al. [37]	173	Higher burnout and PTSD were found in nurses considering a change of career, more frequently screened positive for anxiety and depression, were more likely to respond negatively regarding their team members, teamwork, and impact of their work
		A large portion of nurses with both burnout and significant PTSD symptoms found their symptoms interfered with their work and personal lives
Dos Santos Alves et al. [39]	267	Lower burnout associated with the outcome of positive assessments of the safety climate
Günüşen et al. [48]	117	Secondary traumatic stress (STS) predicted 17% of **EE**
		STS predicted 28% of **DP**
		STS did not predict **PA** significantly
Latimer et al. [58]	27	Higher burnout associated with nurses with higher secondary trauma
Li et al. [60]	251	Higher likelihood of burnout with higher levels of PTSD
		Burnout development secondary to PTSD symptoms may be mitigated by group cohesion
Lin et al. [63]	144	Higher burnout associated with the outcome of statistically significant influence on depression levels
Meadors et al. [66]	23	Higher burnout associated with the outcome of higher levels of compassion fatigue
Meyer et al. [16]	251	Higher burnout predicated by compassion fatigue after controlling for pre-existing stress exposure
		Higher burnout predicated by current stress exposure after controlling for pre-existing stress exposure Mediated association: current stress exposure predicted higher levels of compassion fatigue which then predicted higher levels of burnout after 3 months of bedside
		Exposure
Morrison Wylde et al. [70]	95	Higher burnout associated with PTSD symptoms and compassion fatigue
Moussa and Mahmood [71]	55	Increased **PA** associated with the outcome of an increase in the mother’s satisfaction with meeting the child’s needs and expectations
Skorobogatova et al. [89]	94	Burnout associated with the symptom of tiredness
Tawfik et al. [96]	1 464	Higher burnout found in understaffed units with the outcome of higher infection rates during times when nurses feel overworked (likely when attention to infection prevention decreases)

*EE* Emotional Exhaustion, a subscale of the Maslach Burnout Inventory; *PA* Personal Accomplishment, a subscale of the Maslach Burnout Inventory; *PTSD* post-traumatic stress disorder

### Burnout interventions

Seven of the 78 studies included interventions to mitigate burnout (Table 5). Interventions included coping workshops [42], mindfulness activities [47, 68, 70], workshops to improve knowledge/understanding of their patient population [81, 85], and clinical supervision [50]. Only three of the seven interventions studied provided varying positive impacts on burnout scores [42, 70, 85]. An in-person day-long retreat resulted in a significant improvement in emotional exhaustion for pediatric nurses. The intervention involved didactic and hands-on trauma, adaptive grief, and coping strategies; half of the subjects were also randomized to a booster session 6 months later [42]. Another intervention involved a 90-min interactive module on clinical skills surrounding the management of pediatric pain and resulted in a significant decrease in emotional exhaustion and depersonalization [85]. Finally, the third study of smartphone-delivered mindfulness interventions showed a marginal decrease in burnout compared to nurses receiving traditional mindfulness interventions [70].

**Table 5:** Interventions for pediatric nurse burnout

Author(s)	Number of pediatric nurses	Type of intervention	Result
Edmonds et al. [42]	88	**Care for the Professional Caregiver Program (CPCP)**: day-long retreat, includes didactic and discussion-based coverage of vicarious trauma, loss and adaptive coping with grief. Practical, group-based practice of coping strategies presented such as guided imagery, relaxation, body movement, and mindful breathing techniques that have been adapted for the workplace. Half of the subjects were randomly assigned to a booster session 6 months later.	Pediatric nurses showed greatest improvement in the group in **EE** scores 1 month post-intervention and 7 months post-intervention. The results not impacted by receiving booster session or not.
Gauthier et al. [47]	45	5-min daily mindfulness sessions. Conducted on the unit, as a group, facilitated by a mindfulness meditation instructor. Mindfulness CDs and booklets were distributed after the 1-month follow-up surveys were completed.	1) Intervention was found to be feasible for PICU nurses. **2) EE** was negatively correlated with mindfulness at all three time points. 3) **PA** was positively correlated with mindfulness at all three time points. 4) **DP** was not correlated with mindfulness at time 3 but was negatively correlated with mindfulness at times 1 and 2.
Hallberg [50]	11	Systematic group clinical supervision was performed every third week for two full hours (14 sessions/28 h all together. Supervision performed by a registered nurse, with advanced training and extensive experience in psychiatric care.	The mean score of the tedium degree decreased over the 12 months significantly for mental exhaustion. There were no significant changes in the degree of burnout as measured by the MBI.
Moody et al. [68]	25	**Mindfulness-based course (MBC):** 8 weeks of didactic and experiential mindfulness education via a structured, skills-training course delivered in a group setting at their hospital.; designed and facilitated at each site by a team of two licensed clinicians with extensive training and experience; included journaling.	No significant differences between the groups at baseline or at follow-up on the MBI.
Morrison Wylde et al. [70]	95	**Traditionally delivered mindfulness (TDM):** Nurses in the TDM intervention group (September 2013) received one group session per week for 4 weeks led by a trained Buddhist Priest, taught within different activities. Participants were encouraged to practice mindfulness at other times during the day, but it was not mandatory or assigned as homework. **Smartphone-delivered mindfulness (SDM):** Nurses in the SDM received a free 3-month subscription to a guided mindfulness meditation platform available via website or smartphone application.	SDM group reported significantly more “acting with awareness” and marginally more “non-reactivity to inner experience” skills compared to the TDM group. The SDM group showed marginally more compassion satisfaction and marginally less burnout. The SDM group had a lower risk for compassion fatigue compared to the TDM group, but only when the nurses had previous sub-clinical post-traumatic symptoms.
Richter et al. [81]	17	Nurses helped in the development of intervention materials. Intervention package included five, short educational videos created to demonstrate to nursing staff and caregivers’ solutions to difficulties in caring for hospitalized children affected by HIV/AIDS. Sessions run every 2 weeks.	No changes in nurse well-being were found across the pre/post-intervention phases.
			Post-intervention, patient mothers rated nurses as more supportive; mother-child interaction during feeding was more relaxed and engaged, babies were less socially withdrawn.
Rodrigues et al. [84]	33	**Nursing know-how: skills in working with pediatric chronic pain:** 90-min group session developed from previous knowledge needs assessment (Rodrigues et al. 2017). Modules contained education and case-based role play using nurse’s real experiences.	Significant improvements on both indicators of burnout—**EE** and **DP**—over the 3-month period. However, the proportion of nurses with high **EE** and **DP** is still high.

*EE* Emotional Exhaustion, a subscale of the Maslach Burnout Inventory; *DP* Depersonalization, a subscale of the Maslach Burnout Inventory; *PA* Personal Accomplishment, a subscale of the Maslach Burnout Inventory; *MBI* Maslach Burnout Inventory; *TDM* traditionally delivered mindfulness; *SDM* smartphone-delivered mindfulness; *MBC* mindfulness-based course

## Discussion

To our knowledge, this is the first scoping review that focuses on what is known about pediatric nurse burnout. Burnout was measured with a variety of instruments and interpretations, thereby making score comparisons a challenge. Even in those studies that used the MBI, the most commonly used burnout measurement [105], variations of the tool were applied, as were diverse subscale cutoff scores. Similar challenges in synthesizing extremely heterogeneous burnout data were echoed in a 2018 JAMA review of the prevalence of burnout among different types of physicians [106]. Of the MBI *Emotional Exhaustion* and *Depersonalization* subscale results that were synthesized, the results showed moderate scores indicating a significant level of burnout in pediatric nurses. *Personal Accomplishment* subscale results were high, perhaps indicating a factor of pediatric nursing that increases resilience despite moderate burnout in other domains. In studies that compared nurses who work in pediatric units to other in-patient units, burnout results were mixed [26, 32, 35, 36, 38, 74, 78, 94]. The majority of the included studies identified correlational relationships using cross-sectional study designs, which limited causal inferences. Study designs, such as longitudinal approaches, would allow for causal inference and in-depth analysis of this phenomenon in this unique population.

### Nurse personal factors

Pediatric nurse demographic factors that are associated with burnout, such as age, work experience, and level of education, have been a common area of studied burnout associations across other healthcare populations. Similar burnout associations were found in research studying healthcare providers caring for adults such as younger age (< 31 years) [107, 108] and years of experience (> 7 years) [109]. It is likely that nurses new to the profession are younger, are experiencing the challenges of the nursing profession for the first time, and are less likely to have well-developed skills for resiliency. Given that the start of nurses’ careers is a vulnerable stage for burnout, nursing schools and orientation programs may be well-positioned to highlight burnout prevention and mitigation strategies with students and new hires [107]. Personality traits such as high neuroticism and low agreeableness were found to be associated with pediatric nurse burnout [31]. These results have been supported in other nurse and physician populations, along with conscientiousness, extraversion, and openness contributing to lower levels of burnout [110–113]. Although personality traits appear to have significant correlations with healthcare provider burnout, targeting modulation of personality traits as a mitigation strategy for burnout may be a high-cost, low-yield strategy.

It has been suggested that healthcare provider burnout is not a failure on the part of the individual, rather it is a culmination of impacts stemming from the work environment and the healthcare system as a whole [114]. Responsibility, then, is thought to lie within the individual, the organization, and the profession in general.

### Work environment

Job demands and resource variables in pediatric nursing lead to increased burnout, work-life interference, psychosomatic complaints, and intent to leave; these associations are also represented in adult nursing literature [102, 115], including associations with excessive workload, number of assigned patients, admissions, understaffing, and longer shifts [116–119]. Although Bursch et al. [36] found that pediatric nurses who worked straight day shifts had higher depersonalization than those who worked mixed shifts or just night shifts, Poncet et al. [108] found that working more night shifts was associated with higher burnout in adult critical care nurses. Day shift nurses have potentially more strenuous workloads as patients are more wakeful, have diagnostic tests, or consulting services visiting; however, night shifts could be perceived as more strenuous as it requires the provider to work against their natural circadian rhythm and less support staff are available [120]. These results may also be dependent on individuals’ preference and the specific unit on which they work.

Systems issues such as overwhelming clerical work, administrative, and resource issues have impacts on provider burnout in both the pediatric nurse and general physician populations [36, 56, 65, 121]. Poor leadership is associated with pediatric nurse burnout as identified by Bilial and Ahmed [33] and Druxbury et al. [41]; this relationship is echoed in research with physicians, nurses, and allied health [122]. In pediatric nurses [39], increased perception of organizational support is associated with lower burnout; this association is supported in general nursing populations [119, 123]. In all populations, the support a healthcare provider perceives they get from the organization is predictive of their level of organizational commitment. When healthcare providers perceive that they have high organizational support, they will exhibit greater organizational citizenship behavior, which are extra-role tasks that ultimately improve the organization [124]. Burnout itself results in reduced organizational commitment on the part of the healthcare provider [125].

The experience of witnessing patient suffering and death [65, 122], uncertainty around plan/utility of care [15, 56, 126], moral distress [15, 85], and behavioral issues with patient families (e.g., aggressive patients/families) [65, 79, 127] were found to be significant factors that contributed to burnout in both pediatric nurses and general population physicians and nurses.

### Work attitudes

As might be expected, optimism, self-efficiency, resilience, and positive coping strategies are supported as inversely related to burnout in broader nursing populations [128–130]. The identification and treatment of burnout is particularly important to consider in light of the evidence that burnout is inversely related to job satisfaction and burnout is a contagious phenomenon between nurses; therefore, early intervention is essential to prevent transmission among staff [131–133].

### Work outcomes

The association between burnout and patient satisfaction and intent to leave has been reported in non-pediatric nurse populations as well [5, 6, 115, 134–136]. It is likely that as nurses become increasingly burned out their satisfaction with their jobs decreases and their desire to leave their position increases. This linkage highlights the importance of addressing nurse burnout in the organization to retain staff and reduce the financial and tacit knowledge losses associated with high nurse turnover.

Higher work-related burnout is also associated with mental health conditions such as anxiety and depression in pediatric nurses; this is represented in several studies of other healthcare provider populations [113, 137–140]. However, the majority of these associations are correlational; thus, they are left open for further assessment if they impact the development of burnout or if burnout impacted their development. Further research is needed to confirm causal, directional effects.

The relationship of increased clinician burnout and decreased patient safety has been supported in additional studies of healthcare provider burnout [7, 141]. As clinician burnout increases, the detachment from patients and their work does too, which may contribute to negative attitudes toward patient safety, incomplete infection control practices, and decreased patient engagement [7, 141]. Reducing burnout has the potential to impact patient safety; the Quadruple Aim of Healthcare hopes to improve patient outcomes, such as safety, through the addition of clinician well-being as a primary aim in the model [142].

### Interventions

Although only seven of the studies analyzed in this review included interventions, there is modest evidence on the efficacy of burnout interventions in the broader healthcare provider population. Similar to the results of Hallberg [50], a study of Swedish district nurses showed no impact of clinical supervision on burnout [143]. While Morrison Wylde et al. [70] found a marginal improvement in pediatric nurse burnout with smartphone-based intervention vs. traditional mindfulness interventions, studies investigating mindfulness interventions in other healthcare populations reported mixed results [144–147]. Similar to pediatric nurses [84], social workers showed a significant decrease in burnout after attending skills development courses [148] suggesting that improving clinical knowledge and skills may reduce burnout. This is supported by the finding that pediatric nurses with lower clinical competency and education level have increased burnout [57, 90]. Although Edmonds et al. [42] showed significant decreases in pediatric nurse burnout using in-person trauma, adaptive grief, and coping sessions with follow-up, similar sessions have shown mixed results in other healthcare provider populations [149–151]. More research is needed to identify reliable interventions for pediatric nurse burnout that can be pre-emptively and routinely implemented by nursing schools and healthcare organizations.

### Study limitations

The search strategy was limited to publications in English; thus, potentially relevant studies in other languages were excluded. Gray literature was not included; thus, informal annual surveys conducted at various healthcare institutions may have been missed; however, this was outweighed by the desire to only include peer-reviewed literature to ensure the quality of data reviewed [152]. Third, the definition of “nurses” varies internationally as does their required education and scope of practice; however, the slight variations were outweighed by the need to include thorough, culturally diverse research. Finally, the extreme heterogeneity of the burnout measurement tools and their application and interpretation inhibited the comparison of results across studies.

## Conclusion

Our scoping review showed inconsistent measurement and interpretation of pediatric nurse burnout scores. Factors associated with pediatric nurse burnout were similar to those found in other healthcare professional groups and can be separated into the domains of nurse personal factors, work environment, work attitudes, and work outcomes. Only 45 of the 78 studies reviewed studied exclusive populations of pediatric nurses, and most associations identified were correlational. Few interventions to prevent or mitigate pediatric nurse burnout have been undertaken, and the results were mixed, at best. Further studies using mixed methods are needed to expand on these results and incorporate the direct feedback of the nurses. Additional research is needed to develop and test interventions for pediatric nurse burnout. The improvement of pediatric nurse burnout has the potential to improve nurse well-being and, ultimately, patient care.

## Data Availability

The complete list of articles used as data in this review is available from the corresponding author on reasonable request.

## Abbreviations

MBI: Maslach Burnout Inventory
NICU: Neonatal intensive care unit
PICU: Pediatric intensive care unit
PRISMA: Preferred Reporting Items for Systematic Reviews and Meta-Analyses
PTSD: Post-traumatic stress disorder
